# Silence or Voice? Agency Freedom among Elderly Women Living in Extended Families in Urban India

**DOI:** 10.3390/ijerph17238779

**Published:** 2020-11-26

**Authors:** Ildikó Asztalos Morell, Santa De, Pravina Mahadalkar, Carl Johansson, Lena-Karin Gustafsson

**Affiliations:** 1 Department of Urban and Rural Development, Swedish University of Agricultural Sciences (SLU), 75007 Uppsala, Sweden; 2 School of Health, Care and Welfare, Mälardalen University, 72123 Västerås, Sweden; carl.johansson@mdh.se (C.J.); lena-karin.gustafsson@mdh.se (L.-K.G.); 3 College of Nursing, Bharati Vidyapeeth Deemed University (BVDU), Pune 411043, India; santade.ray@gmail.com (S.D.); 31pravina1965@gmail.com (P.M.)

**Keywords:** ageing, India, women, narrative, subaltern, Hindu, capability, agency freedom, elderly, family care, care regime

## Abstract

The preferential form of living for the elderly in India is within the extended family. India is undergoing rapid economic development, an increase in mobility, and changes in gender norms due to an increase in women’s labour force participation, which places challenges on traditional intergenerational relationships. Ageing and the well-being of the elderly is a rising concern, especially considering that their proportion of the population is expected to grow rapidly in coming decades. There is a lack of universal state provision for the elderly’s basic needs, which is especially profound for elderly women, since most do not have an independent income. This leaves the elderly dependent upon the benevolence of their adult children’s families or other relatives. This paper explores, with help of narrative analysis and critical contributions from capability theory, elderly women’s agency freedoms and how this can be contextualised with their varying capability sets. With help of Spivak’s notion of the silent subaltern, the paper anchors elderly women’s abilities to voice to their agency freedom. The master narrative of *the silent supportive wife and side-lined mother-in-law* as well as three counter-narratives explore alternative agencies taken by elderly women.

## 1. Introduction

Ageing poses increasing challenges for India. The proportion of elderly persons over age 60 grew from 5.4% in 1950 to 9% in 2016 [1] and is expected to reach 20% by 2050 [2]. The absolute number of those over age 60 grew almost six-fold from 20.3 million in 1950 to more than 116 million in 2016, a change primarily attributed to rising life expectancies. The Indian care regime is under transformation. A care regime reflects the differential importance of family, state, market, and civil society in the provision of care for the elderly population [3]. The elderly care system in India has been traditionally home-based and embedded in the intergenerational family system [4] with an underdeveloped service sector and a rudimentary social welfare system supporting the elderly [5]. The responsibility of the family for its elderly is imprinted in the legal system via the Maintenance and Welfare of Parents and Senior Citizens Act of 2007 [6]. However, this system is being partially eroded [7]. Changing family and intergenerational relations, including women’s increased labour force participation, decrease the availability of working-age people to provide care [8]. Increased internal migration as well as international work migration [9] is expected to add to the care deficit [10] and transform the preconditions for care provision. Social security for the elderly reflects the extensive socio-economic inequalities in India. The social/pension security system has not taken on board the vast differences between the elderly (especially between rural and urban), with a large group of very poor, falling outside social security systems, since only 20% of the elderly had a pension providing them with a liveable income in 2003 [11].

Ageing poses gender specific challenges due to the disadvantaged status of elderly women. The overwhelming majority of elderly women (58.7%) compared to 26% of men, do not have any income. Thus 74% of elderly women are fully economically dependent. Only 16.5% of men, compared to 6.8% of women receive a retirement pension from former employment, while an additional 13.7% of men and 22.4% of women receive social pensions which are not meant to fully cover living costs. A large proportion of elderly men (41.5%) and some women (10.1%) have work-related income [12]. Not surprisingly, women in poverty show greater dissatisfaction with life [13]. Ageing poses challenges, not least to health care [1,14]. There is an ongoing feminisation of ageing and with it ageing related poverty [15]. Public recognition, the dominant social imaginary, the master narrative of the elderly, including elderly women, has characterized them as “bereft of agency, autonomy and desire, dominated by frailty and failure” [16]. In India Hinduism influenced the gendered recognition of ageing and has constructed the ageing woman as subservient to her husband and dependent on the benevolence of her children [17]. Elderly women continue to hold the role of caregiver for their husbands [15]. Half (48%) of women over 60 are widows compared to 15% of men and are therefore more vulnerable Elderly women have poorer personal resources and illiteracy is higher among them (71.5%) compared to men (40.9%) [18]. The gender gap in illiteracy prevails even in urban areas. Meanwhile, the proportion of women among those with higher education has grown from 10% in 1950 to 46% in 2016 [19]. Although the positive impact on education on health and autonomy of women is documented [20] we lack information on how education influences elderly women’s health and autonomy.

Despite the growing awareness of the conditions of elderly women [21], there is a continued need to explore the health and well-being of elderly women with different resources within the context of dynamically changing conditions for the provision of care by families [22]. This study aims to fill this gap by giving voice to elderly women with varied social resources and family status in an urban setting.

## 2. Problematising Health and Voice in Later Life

Active Ageing, promoted by the World Health Organization, WHO [23] offers a new social policy paradigm [24] concerning the well-being of older persons emphasizing preventive measures, with the focus on avoidance of disease and disability, maintenance of cognitive and physical functions, and social engagement [25,26]. It advocates the optimisation of opportunities for health, social participation, and security in order to enhance one’s quality of life while ageing [24]. Active ageing has also gained recognition in India. The concept of active ageing is contested from the perspective of how policy measures are translated into practice and how they correspond to socially, culturally, medically diverse conditions of older adults as well as how those receiving care can participate in forming these activities [27,28,29,30,31,32]. Holistic perspectives, such as Nordenfeldt’s welfare theory of health WTH [33,34] associates health to a person’s ability to attain certain vital life goals (VLG) in different areas of their lives. Thus, health is related to self-realisation and well-being [32]. However, even WTH leaves the contextual limitations for individuals to realise their VLGs under conceptualised.

Drawing on Sen’s [35] theory, an individual’s freedom of agency depends upon the agency/capability gap they experience while attempting to achieve those goals they value [35]. For Sen, agency implies people’s capabilities to reach the kind of “functionings” and “achievements” they desire taken the capabilities they have. Achievements are who we can be (beings) while “functionings” are what we can do: (doings). These “beings” and “doings” are ends to achieve. Meanwhile, the means needed to achieve these, depend on our capabilities, which are our possibilities or potentials to actualise them. In Sen’s framework, choice and agency are central. This notion is revised by Hobson [36], who argues that capabilities to achieve valued outcomes such as quality of life are contextual to social institutions and normative structures, some of which enable agency while others constrain the choices and claims (p. 150). Therefore choices (freedoms) are constrained [37].

This study is based on this notion of the constrained nature of choices. Thus, agency is perceived as an exercise of choices limited by “opportunity structures.” Capability framework, using Hobson’s [36] formulation p 149 “is an evaluative space to assess well-being and quality of life and the freedom to pursue it.” Applied to the understanding of how the agencies of the elderly ageing in families are gendered, this study explores how people can realise those goals they value by converting their entitlements to capabilities for a better life in old age. We view these conversions as responses within the given contextual conditions characterising their capability sets that emerged along their life paths and were formed within specific normative and institutional contexts.

An important component of Hobson’s agency theory is the role of societal norms and institutions for agency freedom. This resonates well with Spivak’s [38] theory of the silence of the “subaltern” that places elderly women’s agency in India into the normative context of the Hindu *Ashramas*. The system of *Ashrama* reflects upon the path to death, the relationship between generations and genders and wealth and plays an important role for this normative structure in India. It refers to four ideal stages of the life course [39,40] directed towards different life goals, which are associated with the notion of four essential goals of life [41]. The first stage is that of the *Brahmachrya* (student), followed by the *Grahastha* (householder), *Vanaprastha* (hermit) and then the *Samnyasa* (renunciate). The state of *Vanaprastha* requires the person to withdraw from the affairs of the world [42] p. 88. As a hermit, one should renounce familial ties and social relations, practice “self-restraint, friendliness, charity and a compassionate attitude toward all creatures” [41] p. 184. The goal during the fourth phase, *samnyasa*, is to reach to the union between the person and God through total severance of worldly ties [42] and focus on reaching inner spirituality. These teachings have continued relevance for cultural perceptions of ageing and meeting death and were found to impact on elderly’s life satisfaction and psychological well-being [43].

Meanwhile, in her theory of the “subaltern” criticising the monolithic colonial production of the “third world woman” Spivak [38] also raised criticism against the empty place for criticism of gender asymmetry in the system of Ashrama. The wife of the hermit man might accompany her husband to this state yet has no access to the final phase herself. When her husband dies, she returns to the state of the child. Thus, the “subaltern woman” is silent, lacks voice and operates via the subject status of her husband. Sattee, the self-immolation of the widow had been her only way to release herself from the female body and from the circle of rebirth. Spivak [38] invokes critique against the Hindu concept of the “good wife,” the female infant whose body is imprisoned within the legitimate passion of a single male.

In accordance with Spivak’s notions, a feministic gerontological perspective emphasizes that “giving a voice” to elderly women implies basing social recognition for elderly women on their own experiences and perceptions [44,45]. However, as Fraser’s [46] theory of social justice emphasizes, we need to associate claims for recognition and representation with claims for redistribution and gendered inequalities in older age. Hobson [36], along feministic agency theory, argued that women who lack economic resources do not have the option to protest, i.e., to voice or divorce, which implies: “No Exit, No Voice”. Thus, elderly women’s agency freedom and voice needs to be explored against the backdrop of transitions in women’s status and elderly women’s differing access to resources and care.

As we argued earlier, Indian society is under ongoing transitions changing both gender and intergenerational relations to ageing. Educational and class differences open diverse paths to ageing for elderly women. This study gives voice and recognition to elderly women’s own understandings of ageing while being cognisant of the differences in the redistributive (in)securities characterising their lives. We pursue this goal through the study of discursive struggles over self-construction by elderly women and how these self-construals interplay with the option(s) available to them, taken their capability sets and agency freedoms formed by their personal assets under the dominant conditions of changing institutional and normative realities that their agencies are formed in reference to. Thereof, rooted in the inquiry of the elderly’s health as a function of agency freedom, this study explores whether feeling at peace with suboptimal conditions is the outcome of real possibilities for voicing concerns, or the outcome of accepting suboptimal conditions as the consequence of lacking alternatives for exiting, i.e., choosing otherwise.

## 3. Materials and Methods

### 3.1. Life Story Analyses: A Qualitative Approach

To enhance the understanding of older persons’ agencies, this study relies on the life story approach. According to Yuval-Davis ([46], pp. 201–202), the “*stories people tell themselves and the others about who they are (and who they are not)*” constitute their identity and constitute ways in which elderly women reflect on their lives and their ability to realise themselves through “doings” and as “beings” [35]. Life stories, then, are viewed as self-construals [47] and as stories about a self-realisation process. On one hand, life stories are the products of the person, on the other hand the persons themselves are created through the stories. Thus, the persons are “*both producers and products*” ([47], pp. 15–16). Life story method is appropriate to explore older persons’ freedom of agency since, as Öberg ([47], p. 15) argues: telling a life story can be seen as integrating various life episodes as a: “*reflection over the ‘I’ and over the development of the personality*”, a kind of “*I theory*” and can be scrutinized through an “*analytic net of measuring …. Achieving one’s goals*”.

Life stories are also socially and culturally embedded representations: “*The stories we create with others via socially shared interpretations and evaluations of our personal past constitute our very being*” ([48], p. 55). Memories are embedded in social and cultural institutions and practices. Weltsch [49] emphasises that it is “*individuals as group members who remember*”. Thereof, life stories can be perceived as actualising master narratives that precede us and emerge from transforming society, thereby positioning the narrator within or outside meaningful standard narratives [50,51], also referred to as “*master narratives*,” ([52], p. 3). In India, Hinduism and the *Ashramas* stand in the genealogical foreground for gendered intergenerational relations. To take a Foucauldian stance, the *Ashramas* can be perceived as systems of thought (epistemes) which, as discursive formations, are formative of practices. State policies enforce the extended family as the fundamental institution responsible for elderly care. Meanwhile, counter-narratives emerge in relation to master narratives and define the “*boundaries of the mainstream*” ([53], p. 64), and are formulated within suppressed, marginalised groups. These counter-narratives, or “*storylines*”, allow the individual the opportunity to formulate her/his identity, thereby belonging to a given category or subject position. In times of social transformations, such as transforming family structures, real-life experiences open up arenas for the reconstitution of ideals, and form a dynamic field for potential change and for the reformulation of a sense of identity–“*of who is* ‘*us*’ and “*who is* ‘*them*’” ([54], p. 37).

### 3.2. Data Collection

The study is based on a sample of 6 life story interviews with elderly persons living in families (see Table 1). This sample is part of a larger qualitative study including 41 elderly persons living alone or with their families and 40 elderly persons who received care in government run, government-aided and voluntary institutions in order to obtain a comparative group from a financially less affluent population from the Pune district, in the state of Maharashtra, India.

Semi-structured interviews were conducted with several open-ended questions in the home of the elderly persons. The questions invited the elderly persons to express their perceptions of ageing, how they perceived active ageing, health, care desired and received, and contextual aspects of ageing. Elderly persons were identified via networks of researchers and using non-probability convenient sampling (snowball sampling) technique. Older adults more than 65 years old were chosen for the study. All interviews were voice recorded and averaged approximately one to one and a quarter hour.

### 3.3. Collaborative Research Process and Data Analysis

This study explores voice even in a further meaning. We are cognisant of the complicit nature of giving voice from a research ethical perspective [55]. While Spivak emphasizes the normative gender constraints of the *Ashramas*, she [38] also highlights that it is less important “*Who should speak*” than “*Who will listen*”. As feminist criticism emphasized patterns of inequality result in “*the systematic distortion of some people’s appearance and audibility*” ([56], p. 95). Given the implications of the privileged position of whiteness and academics [57,58] in the politics of voicing, decolonising research has special responsibility for self-reflexive listening. Such reflexivity implies a recognition of the researcher’s positionality in the “*historical structures of privilege and inequality*” [59,60] and how these impact on knowledge production. Therefore, we have applied a reflexive decolonising research methodology, emphasizing listening across difference [61].

The initial phase of the interviews was conducted jointly by a Swedish and an Indian research team. The interview guide was tested by conducting interviews with elderly persons who had a good command of English. The cultural fit of the questions was adjusted. The Indian research team carried out the second phase of the study. Interviews were translated into English. Translations were double-checked by the native speaking Indian project leader. Interpretations were cross-culturally validated throughout the research process. Life story analysis and a qualitative approach [62] were chosen to facilitate reflexive proximity to the lived experiences and self-construals of participants. This proximity was ensured by rigorous inductive coding procedures in combination with abductive analysis.

The analysis started with a review of all interviews. The Indian research team created a summary of each interview containing attribute and structural codes. Responses were then summed up according to key research questions ([62], pp. 69, 84). Based on this first phase, six life story interviews were selected according to marital status and social background. We selected two married women and two widows. Life-stories of the two husbands of the married women were also included. Two of the selected women had no formal education and originated from lower cast and class background and two had higher education and came from higher cast and middle-class families. The selected cases were to be rich in information. Even though qualitative studies do not have the purpose of generalizing their results, one can often transmit qualitative understanding to a wider context even if the number of informants is rather low [63].

In the second stage focus was turned to the chosen six interviews. In this stage a more detailed inductive initial and motif coding [62] was performed, where focus was on identifying turning points in life stories [64] leading to changes in roles and family relations. All six elderly men and women were in the phase of *Vanaprastha* (hermit), although Poormina and Saguna were contemplating the closeness of the final phase, *Samnyasa* (renunciate). All the chosen elderly were Hindu and practiced their religion in the form of prayers in front of home temple, visiting ashrams, participating in religious anniversaries and referring to God as a source of support in life.

In a third stage of analysis, codes representing turning points were further condensed into broader categories and themes constituting storylines. Four main story-lines were identified according to the position of the elderly woman in her relation to her husband, if alive, and to her adult children. In the final abductive stage, storylines were connected to the analysis of capabilities [39]. Among these emerging storylines, we identified as master narrative the storyline of silent supportive wife and side-lined mother-in-law. We also identified three counter-narratives.

## 4. Results

Feminist narrative analysis ([65] highlights the importance of listening, exploring silences and giving voices [40]. In our conceptualization and use of narrative feminist research we are appreciative of intersectionality and explore the interplay between elderly women’s life stories and their resources [66]. Life story method enhances the possibilities of problematising the agency freedom of individuals and allows for contextualising it in relation to their capability sets and opportunity structures [67]. The four narratives (one master narrative and three counter-narratives) discussed below exemplify how elderly women position themselves in the intergenerational family context (see Table 2). Their self-positionings reflect differences in their identities and agencies as well as upon how the agency freedoms of these elderly women are conditioned by their capability sets composed along with intersectional inequality structures.

### 4.1. The Master Narrative of the Silent Supportive Wife and Sidelined Mother-In-Law

According to the fieldnotes taken after the interview with F1 and M1, the interviewed couple are originally from a small village, but lived for 40 years in Mumbai. At the age of 70 they moved to Pune in 2012 to live together with their two sons, two daughters-in-law and four grandchildren. They follow Hindu religious practices. They live in a house. The land is in the husband’s name, and the house has been built by their sons. They live in a partially constructed house which is situated in a semi-urban area in Pune. The house is small for a family of 10. The elderly couple lives on the upper floor in a partially constructed place which only has a roof and no walls. There are two rooms on the ground floor which is occupied by their two sons and their families. They share a common kitchen and bathroom, which is situated at the ground floor. The elderly couple climbs the stairs to the upper floor only at night when they go to sleep. F1, the wife, is 70 years old. She looks unkempt, like she has just woken up from her sleep. She seemed unwilling to express herself openly. She only agreed to give the interview on her husband’s insistence. She was constantly saying that “*I don’t have anything to say, my husband has told you everything*”.

#### 4.1.1. The Double Silence of the Wife

F1, the wife came from a simple background: “*we would do some odd jobs to fill our stomachs…. We used to collect waste materials and sell it off to the recyclers*.” She has not been educated. She asserts to be satisfied with her current state of life: *“I feel good. Nothing else”*.

It seems that her daily achievement of being able to take care of her and her husband’s bodily needs correspond with what she values in life, her vital life goals. She values not being dependent on others help: “*Why would I need anybody’s help? We can do all our work by ourselves.”* She finds that doing the household chores of cooking, washing their clothes, helping her husband, are chores one needs to do and it is a natural: *“No, we should do our work on our own. It is our work we have to do it. Cooking 2–3 rotis is not much work. Once it is finished the work is done. What to do…”* Neither does she complain about eating only one meal a day: “*We don’t have breakfast, so we only cook for one time. I make bhakri once in a day that’s enough for us*”.

Inquiring further, she explains that she does not have any leisure activities, she does not leave the house, only seldomly visits the temple during major functions: “*I will go and bow my head before God and come back. What else to do?*” Her life is conducted in the close confinements of the house. She is similarly content with not having own personal income or owing a telephone. The lack of personal property or income implies that she cannot engage independently in functionings that require money to buy or being able to communicate independently. She denies having the desire for independence and seeks to assure the interviewer that she is content with her state of affairs. Neither she nor her husband participate in shopping for the household: “*we are not involved in buying things for the house.*” This disengagement is also reflected even in her lack of interest to learn new things.

In a contrasting way, her husband, M1, who has been a musician of a traditional instrument, explains, how he maintained his contacts in Mumbai, and is called time to time to play in concerts. He still desires to learn and develop his musical skills. After suffering a stroke, he is being taken care of by F1. He has also access to some income from these occasions and has a mobile phone.

F1 gives the impression of a life lived in the shadow of the family of her children: “*we do our work after they leave.*” She is adjusting her activities in a way so she would not interfere with the activities of the adult children and their families: “*I don’t do anything to anybody. I only do my work. I don’t interfere in anybody’s work. I let them finish their work, till then I relax.”*

She returns, saying throughout the interview that she hasn’t much to add: “*what to say*? She describes how matters in the house are taken care of without asking for her needs: “*They [the adult children] get things for themselves according to what they feel is right.”* Thus, forming an opinion about what is the everyday needs of the grandparents is taken over by the adult children. She feels that making a complaint would discredit the trust in their children’s conformance to the requirements for “goodness” for caring children and would imply distrust and bad feelings: “*They are good. They go about their work. Why should I nag them? They are feeding us well. Why would I nag behind them?”* This statement acknowledges also that their children do not break against moral obligations to secure the well-being of their elderly parents by prioritising work. Work is part of tasks for a good householder.

While F1 denies her need to voice personal wishes and meanings, she admits that her children ask the opinion of her husband, M1, on matters.

*I: do your children ask you for suggestions?*

*IP: They ask their father.*

*I: Do they ask your opinion?*

*IP: They don’t ask me. They ask their father, that’s enough. What do I have to say? I don’t have anything to say. They see what is to be done. What should I do?*

Thus, even if she confirms the moral virtues, i.e., the goodness of her children, since they “*see what is to be done*” without her needing to point it out, the children do ask the opinion of her husband. Thus, she acknowledges the distinction between the normality of her silence and the need of her husband to be heard and consulted on family matters. In this sense, her silence is double: she has no voice direct to her children, and she is to adhere to her husband as for voicing matters from the point of view of elderly parents.

This double silence exemplifies well the gendered expectations on the elderly wife following her husband to the phase of *Vanaparstha* (hermit) exposed by Spivak [40]. While the head of household resigns from the state of *Grahastha* (householder), the wife can follow him to the new state via performing continued service and subordination. Taken the context of the extended family, she endures double subordination due to the silences of the old woman in relation to the family of her adult children.

Inquiring about the relation to her husband, F1 confirms the same appeasement: “*We stay together nicely. Do we have any other option other than staying together?”* Without denying the possibility that this appeasement relies on genuine harmony, we can identify two foundations for appeasement. One would be a kind of moral quest for being good and treat people as you want to be treated by them, as she formulated: *“If we are good to others, others will also be good to you. Why should you fight or argue? You should talk nicely and live peacefully.”* Yet, as another revealing aspect, she highlights the lack of options: “*Do we have any options*?”

As mentioned above, F1 motivates her appeasement with normative rules of goodness, while she finds that the institution of family care is the only option available for them. Since they have moved to Pune, the residence of their adult children, from Mumbai, where they spent their lives as *Grahastha* (householder). They have only their children to rely on: “*In our bad time our children will be there, who else would see us*.” They need to rely on their children both in terms of future care needs and finances:

*I: Aha. Do you have any money for your personal use?*

*IP: (Shaking her hand). All that [money matters] are taken care of by our children. We don’t get involved in all that. … it is only they who see what is to be done.*

*I: Do you feel that is alright? Do you feel you should have some money with you?*

*IP: We get food to eat, that is enough. What do I have to do with money and all? (F1)*

#### 4.1.2. The Respected Voice of the Male Hermit

The way how F1’s husband, M1, represents his life and position in the family is the opposite of F1. M1 presents himself as a respected artist, who is still invited to Mumbai to play. Due to his artist background, he maintained, unlike F1, connections with the community that gives him pride:

*“I keep meeting people from my community and people from my profession. …They have been good to me since the beginning… Big artists call me… Big artists respect and felicitate me… I have very good relations with them...I have received love from them. I have lived my life happily till now.” (M1)*

His engagement provides also some income, which the couple uses on medicine. Sometimes he can help out his children with the money. Concerning household duties, he claims: “*I don’t help in doing household work*” and that his *“daughters-in-law do the cooking... They buy the groceries in the house. …They do everything. We absolutely don’t look into anything… They take care of everything…*” This leaves the cooking, cleaning and caring of his wife for them two invisible.

Although, due to his stroke he needs support in physical activities, he is emphasising his own efforts to do things by himself: “*I don’t need any help... But I am old… once my children go to work... my wife heats the water for bathing. I do as much as I can do by myself”* and acknowledges only reluctantly the support given by his wife: *“In case anything unwanted happens my wife is there... My wife supports me... I hold my wife’s hand... take a little support of the wall.*” Reflecting on emotions, in contrast to F1′s denial of negative emotions, M1 admits that “*sometimes we get angry… we do not talk to each other.*”

As F1 asserted, M1 has, in his own perception a strong voice in the family. He asserts the good quality of relation by the younger generation listening to his advice:

*“My relation is good with my sons… they listen to me... sometimes I get angry… still they listen to me… Even though I have become old… Even though I may say things they don’t like… they bow their heads and listen to me… nobody back answers me…Neither my daughters-in-law, nor my grandchildren, nor my sons… none of them give back answer…. My grandchildren are also good. They also listen to me. … Whenever I can I guide them. They also do as I say...” (M1)*

In return he is counting on the support of his children in need: “if I need some support then I will tell my children… And my children also support me… Even if they are facing problems… they adjust for me.” Neither F1 nor her husband receives a retirement pension and they lack an independent source of subsistence. That they “are not involved in money matters” has to do with not having any spending money from their children. This lack of options and total dependency on their children in terms of financial matters and care is interpreted as a key source for resignation from desires, characterising the older wife of the retired male Vanaprastha (hermit).

#### 4.1.3. Two Life Stories, One Storyline

This master narrative, represented by the intersecting life stories of F1 and M1, expresses the gendered model for rooted in traditional Hinduism. As Spivak [40] highlighted gender contract built on the four Asanas, constitutes the wife as an eternal supporter of her husband, even in his retirement. The wife’s unwillingness to talk and appeasement with her role without contest elucidates her silence. As her self-reflection on lacking alternatives as the reason for her appeasement with the state of affairs well illustrates, her agency freedom, according to the emphasis in capability theory [39], needs to be understood in relation to the deficiencies of her capability sets. She lacks both personal resources or institutional alternatives to do otherwise. Thus, her acquiescence and silence can be seen as an acceptance of what she cannot change, and the lack of exit as an option. These two parallel life stories illustrate the gendered inequalities of silences that underline the final stages of life, offering voice and showing respect to older men, while a lack of acknowledgement and silent service for elderly women.

The narrative of the traditional extended family living with the silence of the wife can be considered as a master narrative in the Indian context. Based on the analysis of the interviews of elderly women living with their children competing counter-narratives reflecting emerging alternative life trajectories emerged. These counter-narratives offered different degrees of agency freedom for the elderly women depending on differences in their capability sets. The following key factors that emerged in the course of inductive analysis of the interviews emerge influencing elderly women’s capability sets: the level of their formal education; the presence of previous experience of independent provision; entitlement to pension or other material or immaterial resources; the residence being in the families of their sons or daughters; the permanency of the stay at one child or several children; and the status of their adult children. Based on the combination of these, three counternarratives representing alternative positionings of the woman were identified.

### 4.2. Counter-Narratives of Gendered Care Provision and Elderly Women’s Voices

#### 4.2.1. From Self-Reliant Widow to Mobile Grandmother

While some key elements of the narrative of F4, a widow since the early ages of her children, reminds on the master narrative, the life story of F4 differs in some important aspects. She lives in a system of shifting residences at three different adult children: 5–6 months at her two sons and 2–4 weeks at her daughter’s family. She has, similar to F1, no formal education, and, due to the early passing away of her husband, she was forced to rely on her own powers to provide for her children (even if assisted in some ways by her husband’s family). She has accomplished this via different micro business activities. She has a natural curiosity and has vivid social networks, primarily in the city of her older son, but also at her younger son. This gives her a life offering joys outside the family of her children. Nonetheless, her relation with her children resembles in many ways that of F1. She steps back, especially concerning the kitchen sphere, and is very definite about not intervening in the spheres of her daughters-in-law. When asked if she makes any request to assist with the food preparation, she is resolute:

*“No… daughters-in-law do everything. Why should we do? They make…. Prepare everything of our favourite for me without my demands. So, why should we say that I want this and that… No… no… they don’t ask me to cook. She prepares spices… what she likes. (F4)*

She is careful not to voice wishes. Even if she appreciates if relatives ask about her needs. Her account of an encounter with a well-to-do nephew illustrates this point:

*“They used to say me Kaku (aunty). … they will talk nicely, they will greet us. … he immediately comes and says aunty please come… he sits with us… chit-chat with us. He always asks, aunty do you need something? I never ask for anything. But he asks that is admirable, isn’t it? (F4)*

She accounts of being respected, yet, perhaps resonating with the traditional moral code, she keeps from voicing desires. Even if, similar to F1, F4 has no pension or income, she receives bi-monthly contribution from her sons, both of whom got good education thanks to her efforts, who also provide for her medicine. While her children demand that this allowance is spent on her personal needs, she uses most of the money to offer small presents among her relatives.

*“We have to give… we have many relatives, so if they come then we have to give at least Rs. 100/- to them… My granddaughter has married before 2 years. So, whenever she comes I was giving her money … for Ganesh festival. … we gave strainer …We have to give at least Rs. 100–100… … I give Rs. 100/-to everyone there… Everyone shouts on me that why are you giving money to others. We give the money to you. My daughter also scolds me… she says I should not to give money … everyone says … I will give to everyone. Everyone is equal for me…” (F4)*

As this story illustrates, the small allowance opens opportunities for her to engage in social life, and assert herself as part of social interactions according to her choice offering a window of opportunity for resistance and voice. In contrast, she elucidates how being deprived of money made her feel vulnerable, when, as young mother, she had to stay with her son undergoing an operation in a hospital without a penny:

*“I spent my worst days there. …through someone my mother-in-law were used to send tiffin… so, I was getting tiffin at 4pm… up to that time I was not eating anything. Because at that time husbands were not giving you money… he never gave money. But the ladies in that hospital were nice…. Some of them were Gujarati… some were Marwari… they used to give me tea.” (F4)*

Compared to F1, F4’s capability set is enriched with an entrepreneurial spirit with ability and habit of engage with the surrounding world. She has also children who are better off financially and can offer her a small allowance that she is free to dispose over. Meanwhile the living arrangement of moving from one child to the other creates its own risks. She has better access to medical care at one of the sons, which makes that she does not obtain proper medical attention for an infection for her eyes.

#### 4.2.2. Towards a Collaborative Extended Family Model

The case of an educated couple provides a life story articulating a counternarrative, where, unlike in the case of F1 and F4, the relations to the daughters-in-law seem to be more on a collaborative basis, than along hierarchical distinctions. F2 and M2 are both educated with state pension over 20,000 rupees a month. They have two married sons who moved to Pune when they obtained good jobs, with the precondition that the retired couple would move after them. They feel strong familiar attachment: “*there is no point to live alone without children. I cannot live without my son*.” Wanting to support their son in his carrier, they bought a flat in Pune 10 years ago, where they live now. They live together with one of the sons and his wife, both of whom have a university degree, and their grandchildren. Their other son has been working abroad for three years. They bought a flat for him also. When both sons are settled in India: “*they both are telling that both of us want the parents*.” She would not want to move to old age home since, as she states *“I am very confident that … my sons will look after us.*” even when they would require more assistance.

Unlike F1 or F4, M2 has more of a collaborative relationship with her daughters-in-law. She has taken early retirement when one of her daughters-in-law delivered her first baby, in order to support her. Living now with the other daughter-in-law, she explains that she is doing most of the work in the kitchen:

*“I am the first to wake up in the family, 6 o’clock in the morning, practice some yoga, and then prepare tea and breakfast for everyone. I love feeding everyone. I make tea and all breakfast to dinner. Breakfast is important.” (M2)*

She is proud of her daughters–in-law, who both are educated: *“both of them are very nice. They are like my daughters. They are good girls.”* She receives help with the household work from her daughter-in-law as well as has a financially compensated maid.

The younger generation is respectful: “*They ask for guidance. And we are also happily advising them.*” But they are given the opportunity to decide how they would like to furnish the flat: “*children ask for advice but we also leave it to the children to decide.”* They perceive the habitat as joint: “*It is not my house only, it is their house also.”*

Moving implied even for this family a loss of previous social network and relatives: “Pune is a big city with all kinds of people. There is no such an attachment between people as in A. My friends are there. Close friends.” However, they can afford regular visits and have a small apartment in their hometown. Also, M2 started new hobbies and engagement with cultural activities and created a rich social and cultural life for herself in Pune.

F2, M2’s husband, has been a university teacher with a substantial pension. He is appreciative of his wife’s professional and artistic accomplishments, and values her contributions to the household. He contributes to the domestic housework:

*“we both are cooperatively doing … all the things. Sometimes I also participate in cooking. Participate in cleaning… in housekeeping. We both do it. I am not only sitting… or only ordering [laugh]” (F2)*

He is hiring maids to assist M2, since F2 finds his wife to work too hard in the household. He, as M2, would also like to get support from his children when becoming frail and stay within the extended family, yet thinks that hiring a maid can serve as an alternative source of help.

Thus, a strong capability set, high level of education, good pension, cultural resources and social networks, allowed M2 to create for herself new “doings” and “beings” after moving to the extended family residence, since she developed herself to an artist upon retirement, that fulfil her vital life goals, even if her sons’ movement obliged her to make sacrifices. She is positioning herself as part of a collaborative extended family model and as an equal partner both to her husband and to her children an image enforced by her husband. Meanwhile, this equality is supported time to time by the assistance of paid maids.

#### 4.2.3. The Professional Grandmother Ready to Break Traditions

*The third counternarrative* is represented by the life story of an educated widow, F3, who similar to F4 circulated her residence between her children. Due to her injury, she has been residing at one of the daughters the past years, who is a nurse and lives close to the hospital. She has only daughters, and moving in with one’s own daughters might open up other kinds of agencies and positionings for elderly women compared to moving in with sons and daughters-in-law. F3 has been educated as a nurse and midwife and ended her carrier as the principal of a nursing school with a nice pension of about 20,000 rupees. For her, professional life had been important. As a young mother, she had to make use of day care facilities, in order to be able to combine work and family life:

*“When I was working, I kept my children at the day care group and play group. I kept them there and did duty…. They agreed to that. They did not get the best food whenever they wanted it. For eight hours no food. I came back and then fed them.” (F3)*

She presents the fact that she as young mother used services to replace her in care duties, as a motivation for her to accept care solutions beyond family provision. Considering the condition of her health and the need for care, she would prefer to reside in an old age home that would be attached to a hospital. As she claims, if an: “*old age home is attached to the hospital, that will be better for people like us to stay there.*” However, those functioning in India typically require family care for sick elderly persons: “*What happens if somebody is sick, the old age home people inform the children [of the elderly] and … the children come and take [them home].*” She sees a growing need for old age homes which can also provide medical care, due to, beside other reasons, the growing international mobility of the younger generation.

In India, families have a moral obligation to take care of their elderly family members, and not doing so is associated with shame. However, due to her own use of daycare services, she sees no reason for her children to feel bad:

*“Now in my old age if this comes I have to accept it. I should not feel bad. And if I join my age group I will be happy. If you find such old age homes [with medical facilities], you may send me there. Then I will adjust there. And for that, I don’t blame anybody, if parents are kept in old age home. Neither should a bad name come on my children. But somebody has to start it. We should go there willingly” (F3)*

Her primary worry is her deteriorating health and increasing care need. Increasing care needs would place too big a burden on her professional daughter: “*it will be very difficult for her to manage children, and do service and me also.*” She desires to go to an old age home also to avoid becoming a burden for whom the family needs to make a sacrifice: “*they cannot leave me alone here and they cannot take me also along with them. How long do they have to sacrifice like this*?”

As of now, she can contribute, even if she is confined to the home, since she cannot go out on her own. Her daughter is a professional, just like her, who has to juggle with work and household responsibilities. She is taking over cooking chores after her daughter, son-in-law and grandchildren leave. “*Morning cooking is little bit in a hurry, busy time, so at that time I stay away. I don’t disturb them. After that slowly I do other work in the kitchen. So much other work is there after that. I do all that*.” To be able to contribute is what makes her happy: “*If you can help somebody … I feel very happy... I am still useful. Otherwise I cannot do anything without support or burden on somebody. Here I can do something, this much work I have done, I am happy.”*

She would like to move, while still fit: “*so now I have the energy to adjust there, I can remain there*.” With her experience as a nurse, she would like to move to an old age home while still able minded. She would like to contribute to their activities as a nurse. She illustrates it with the example of a hospitalisation, when she ended up helping student nurses with band skill: “*I said … I will teach you. So, the students learned band. There are different patterns to do it … I said this is not the way, follow the rules.*” She imagines that in an old age home, she would also enter the kitchen and take on duties she is qualified for, such as supervision of e.g., hygienic standards.

Thus, unlike F1 and F4, F3 is physically confined to stay in her daughter’s home. Despite her poor health, she has important resources, in form of formal education and previous professional experience, as well as a pension giving her financial resources. She is taken well care of by the family of her daughter, and she still has an independent voice, formulating alternative visions for the remaining period of her advanced years. What seems to hold her and her family back is the strong moral shame attached to a move to an old age home. Adult children are blamed for families not taking care of their elderly family members. She is formulating a powerful counternarrative: “*Then I will adjust there. And for that I don’t blame anybody, if parents are kept at old age home. Neither should a bad name fall on my children.”*

## 5. Discussion

In common with the nature of qualitative studies, there were a limited number of informants in this study. However, the informants provided several varied statements thanks to their willingness to narrate fervently their life stories. Data can, therefore, be considered rich in explicit experiences because of the large amount of text describing the phenomena of interest. As life stories are part of a collective narrative [51,52] rooted in societal norms. Since the narrator positions her/himself within or outside dominant master narratives of their time and place, we can learn about the broader context characterizing the norms and values of a society beyond what the virtues of individual life per se can convey. Thus, the generalizability of the results lies to a large extent in our ability to identify which kind of contextual conditions characterized the life experiences of the elderly women in our sample.

The four life stories chosen to represent the master narrative and three counter-narratives highlight the narrative expressions for the changing identities and agencies of elderly women living together with the families of their adult children. These life stories represent different ways how elderly women position themselves in different types of extended families, an institution that has traditionally been and is still the normative and by law sanctioned form for the care of older persons in India. The analysis highlights elderly women as not simple victims, but as active agents positioning their identities and practices in relation to their capability sets and desired life goals, whether they confirm the master narrative of care by adult children or take a position within unfolding variations of counter-narratives. The storylines relate also to the differences in these elderly women’s life situations (two are married and two are widows), educational and class background (two have a lower-class origin and have no formal education while two of them have university degrees). Three of them have health problems common with their age, tending to have increasing incidents of poor health, while one has become disabled in movement due to an accident.

The life stories represent different types of responses to ageing relying on different types of capability sets, manifesting different functionings and achievements and different agencies of freedom. As the two examples of low educated women highlight, lack of previous pension granting employment as well as lack of pension means dependence on a main male breadwinner and later on adult children. A paucity of state institutions to compensate for lack of independent incomes for the elderly, especially women, strengthens their vulnerabilities.

Reflecting on the dilemma raised by welfare theory of health claiming that health and happiness can be measured by how an individual can fulfil her/his vital life goals [33,34] the acquiescence of F1 with her lack of choices could be perceived as a sign of good health. Taken from the more critical perspective of capability theory [35,36] one would argue that health and happiness assumes agency freedom. And agency freedom assumes that persons possess basic capability sets enabling them to formulate and reach alternative goals. Appeasement with fate due to a lack of any alternatives, thus, is acceptance without real agency freedom. This cannot be a sign of holistic health in a critical meaning. Agency freedom would assume the strengthening of the capability sets of disadvantaged groups, offering entitlements that could enable them to formulate reachable alternatives. Without agency freedom, the ability to choose voice or exit, rather than silent acceptance [36] is hampered.

The two women without formal education differ in the degree of their silences. In contrast to the case of F1, who refrains from voicing concerns both towards her children and her husband, F4, as a longstanding widow, acts as a provider for her family. Thus, she has no husband to subordinate herself to. Despite the accomplishment of raising and educating two of her children, in the families of her sons, she does not make her voice heard on household matters. Nonetheless, the regular contribution of her sons with some allowance gives her the opportunity to dispose of the sum and convert it into small presents with which she can participate in chains of reciprocity of gifts, giving her a sense of meaning and dignity. Thus, the transubstantiation of capital between immaterial and material assets contributes to raising her capability assets [36] and agency freedom.

The case of F4 is similar to the case of F2 and M2, as well as in some respects to F3, as travelling grandparents, they take turns in residing at different children’s homes. This is dissimilar to F1 who resides under the same roof as both of her sons. The travelling grandparents model has the benefit of sharing burdens. However, from the perspective of the elderly person, there are several draw-backs. It destabilises their social networks, access to cultural activities and most importantly, access to key medical services. F4, for example, had her eyes operated on while residing with one of her sons. Post-operative treatment had to be postponed several months until it was time to move back to this son. Access to professional medical care becomes central for the older the person becomes and the more complex their medical needs. This led F3 to stay at one of her daughters, who herself is a nurse and resided close to a hospital where medical treatment was accessible.

The elderly women with university education show more ability to voice concerns both in relation to their adult children and in the case of M2 in relation to her spouse. Thus, they negotiate their options. Having independent pensions as well as life savings in the form of jointly owned real estate, in the case of M2 for example, offers capability sets that can open for agency freedom in decisions. Such freedom is infringed when state institutions, such as healthcare or elderly care with healthcare facilities cannot provide safe alternative solutions. The lack of a pension, accumulated assets, or independent income leaves women with lower levels of formal education locked up in the system of family-based provision of care.

The institution of family-based care is shown as very strong in these cases. Assurance of the “good son and daughter-in-law” as the primary provider of care who is believed to be there when older persons no longer can look after their primary needs is the foundation of being able to believe in safe ageing. The moral righteousness of children is ensured even where the physical conditions under which elderly people live are not optimal and the older parent is left without a penny to dispose over. Even if no physical abuse has been noticed or reported by F1, the deep silences of this old lady point to vulnerabilities. The assurance of basic income for older persons, even those without state employment could improve basic freedoms of older persons to decide over and have unmitigated access to elementary basic resources.

Before such reform is within reach, NGOs and state services catering to the needs of old people would need to identify old persons without social networks, who cannot participate in health promoting activities due to lack of resources and social networks. The increasing social and geographic mobility that Indian society experiences is going to lead to an increase in the number of those older persons that need to follow the families of their children and break up from their settlement of origin. This implies the loss of vital social networks. Older persons can hardly recover such losses due to fading vitality and or independent financial resources. The number of older persons who choose not to live with their adult children due to their fears of becoming isolated and living on their own apart from their children increases [68]. Further research is needed to map the needs of older persons who move with their mobile children to inform social works with older persons.

Finally, the case of F3 illustrates well, how many older persons have vital power and knowhow that society can make use of. It is also important to take on board their visions of how old age homes of the future could look like. These older persons fear idleness and envision old age homes offered opportunities for their social and professional engagement.

## 6. Conclusions

The concept of active ageing has been both valued and criticised. This study confirms the criticism pinpointing the need to anchor active ageing measures to the societal context of elderly persons. Nordenfeldt’s [33] welfare theory of health informed this study of how to understand “health” as a person’s ability to realise vital life goals. Meanwhile, this study highlighted the need to contextualise the embeddedness of vital life goals into norms and problematise what Sen [35] referred to as agency freedom. The critical extension of capability theory [36] allowed us to elucidate how the range of potential vital life goals are formed, dependent on the horizons available for elderly women. Thus, a genuine choice of accepting one’s conditions in life assumes the ability to voice and exit, alternatives that are not given for those elderly women without independent resources. Connecting to Spivak’s [38] notion of the silence of the subaltern, the study showed how lack of voice of elderly women is impinged within the institution of deprivation from independent resources characterising the traditional extended family care model with strict gender and intergenerational hierarchies, with the elderly mother (in law) at the bottom of the scale. Meanwhile, by using narrative analysis, the study also elucidated how elderly women with diverse immaterial and material resources manage to transform the institution of extended family care. They position themselves within counternarratives renegotiating the traditional vertically constructed master narrative of traditional extended family care rooted in the gendered genealogies of the *Ashramas*. Thus, narrative constructions of vital life goals form and are formed by conditions of life and capability sets, facilitating the realization of “beings” and “doings” of elderly women. The life trajectories explored in the dynamic between the master and three counter-narratives reflect well the social and cultural transitions in the role of women and family structures discussed earlier in this study. Among others, it has shown the importance of education and independent resources for women in older age and how it influences their agency freedom and possibilities for voice.

## Figures and Tables

**Table 1:** Interviewed elderly living with their adult children in this study.

Name	Age, Gender and Marital Status	Education and Former Occupation	Family Composition	Living Arrangement	Economy
Gangubai F1	70 female married to Dhondiba	No education, housewife & garbage collector	Living with husband and the families of two sons	The couple lives in the unfinished roof, below the sons in two rooms	No pension, no money
Dhondiba M1	77 male married to Gangubai	Education till second standard, musician			Small occasional incomes to cover medicine
Makarand F2	65 male married to Asawari	PhD degree teacher, state employee	Lives interchangeably with the families of sons	Apartment jointly owned with his wife shared with one son	Good economy with state pension
Asawari M2	67 female married to Makarand	Bachelor degree, state employee			Good economy with state pension
Poormina F3	83 female widow	Bachelor degree nurse and midwife	Lives with the eldest daughters’ family	Lives in daughter’s flat	Good economy with state pension
Saguna F4	78 female widow	No education, small entrepreneur	Lives interchangeably with the families of sons	In children’s flat	Poor economy, no pension, allowance from children

Interviews were conducted in the district of Pune, Maharasta State, India. All names are fictive.

**Table 2:** Master and counter-narratives and belonging storylines.

Narrative Types	Thematic Storylines
The master narrative of the silent supportive wife and side-lined mother-in-law	*The double silence of the ageing wife* *The respected voice of the male hermit* *Two life stories, one storyline*
Counter-narratives of gendered care provision and elderly women’s voices	*From self-reliant widow to mobile ageing grandmother*
	*Towards a collaborative extended family model* *The professional grandmother ready to break traditions*
